# Food packaging’s materials: A food safety perspective

**DOI:** 10.1016/j.sjbs.2021.04.047

**Published:** 2021-04-24

**Authors:** M.S. Alamri, Akram A.A. Qasem, Abdellatif A. Mohamed, Shahzad Hussain, Mohamed A. Ibraheem, Ghalia Shamlan, Hesham A. Alqah, Ali S. Qasha

**Affiliations:** Department of Food Science and Nutrition, King Saud University, P.O. Box 2460, Riyadh 11451, Saudi Arabia

**Keywords:** Food packaging, Chemical contamination, Migration, Food safety

## Abstract

Food packaging serves purposes of food product safety and easy handling and transport by preventing chemical contamination and enhancing shelf life, which provides convenience for consumers. Various types of materials, including plastics, glass, metals, and papers and their composites, have been used for food packaging. However, owing to consumers’ increased health awareness, the significance of transferring harmful materials from packaging materials into foods is of greater concern. This review highlights the interactions of food with packaging materials and elaborates the mechanism, types, and contributing factors of migration of chemical substances from the packaging to foods. Also, various types of chemical migrants from different packaging materials with their possible impacts on food safety and human health are discussed. We conclude with a future outlook based on legislative considerations and ongoing technical contributions to optimization of food–package interactions.

## 1 Introduction

Food packaging is used for diverse products, and food protection along the supply chain is largely based on the packaging (Brody et al., 2008). Without packaging, the handling of food products would be costly and inefficient (Robertson, 2006). Packaging also provides consumers initial product identity before deciding whether to purchase it. Also, consumer demand is changing and now includes such diverse packaging as active and intelligent packaging. These packaging systems interact and respond to the food-packaging environment, where they release some substances in or scavenge some from the packaging headspace and prolong the shelf life of food products (Robertson, 2006). Such innovative packaging is practiced in part to boost sales in a competitive environment. The packaging style and design may also enhance the product’s image and acceptability. Thus, the selection of packaging material is a consideration for consumers at the end of supply chain.

The major objective of packaging is to protect and preserve foods from possible physical, chemical, microbiological, or other hazards that ultimately can impact their quality and safety (Lee, 2010). In the prediction of food shelf life, the design of food packaging is the main consideration. When selecting packaging materials, many factors should be considered, including cost, quality of products, and their ability to maintain product freshness. A few common materials used in food packaging are plastics, paper, glass, and metals. Among these, a wide variety of plastics are used in rigid or flexible food packaging. Packaging materials now include laminates, which were developed by systematically integrating materials with different inherent properties to improve the functionality of the final material. Diverse food packaging and container types are shown in Table 1. In general, various chemical substances are found in foods during different phases of the supply chain; these include micronutrients, flavorings, antimicrobials, antioxidants, pesticides, and mycotoxins. Also, additives such as plasticizers, monomers, and oligomers found in the packaging materials could transfer to the foods upon contact during processing or packaging; this transfer of chemical compounds between the food and packaging is termed “migration” (Arvanitoyannis and Bosnea, 2004). This interactive phenomenon could result in alterations in the quality and also the safety of the food, and flavor may change owing to sorption of aroma and the transfer of undesirable components from the packaging material to the food. Understanding the migration mechanism is crucial for estimating food deterioration when using synthetic polymer-based packaging. However, direct interaction between food and packaging is not necessarily detrimental, as the same principles that because unwanted interactions may also result in desirable outcomes.

**Table 1:** Food packages and container types (Shaw, 2013).

Packaging type	Products type	Application*
Aseptic processing	Egg (liquid/whole) and dairy	Primary
Bags	Potato chips, apples, rice	Primary
Cans	Soup	Primary
Paper (cartons, coated)	Eggs, milk/juices	Primary
Flexible packaging	Bagged salad	Primary
Trays	Meat/fish pieces	Primary
Corrugated boxes	Cereal carton boxes, frozen pizza	Secondary
Pallets	Series of boxes on single pallet for carriage from producing plant to distribution station	Tertiary
Wrappers	To wrap boxes for transport	Tertiary

* Primary packaging is main package used to hold food being processed; secondary packaging combines primary packages inside one box; tertiary packaging combines multiple secondary packages into one pack.

An example of beneficial migration is the oxygen-scavenging films that directly absorb oxygen, prevent microbial growth, and remove undesirable flavors by sorption (Hotchkiss, 1997). The mass transfer has been variously described as a physical interaction in which chemical transfer occurs at the food–packaging interface, a chemical interaction possibly resulting from the corrosive action of food components on metallic packaging, or microbiological food contamination caused by contact with contaminated packaging material (Lee et al., 2008).

Because the interaction between packaging material and food is influenced by many factors, a careful selection of packaging material is required to avoid negative effects on the quality, safety, and shelf stability of products. Product considerations should also include flavor sensitivity, color changes, and microbial activity. To design a suitable food-packaging system, type of polymer, method of preparation, and polymer content-to-food ratio are assessed to help define the interaction level of the food and the package. Also, processing methods as well as time and temperature during food storage should be considered (Hotchkiss, 1997).

### 1.1 Food interaction with packaging materials

The interaction between food and its packaging is a crucial consideration, especially when the food comes in contact with the packaging material. It is during this contact that the intrusion of gases and volatiles, moisture, microorganisms and other low molecular weight compounds occurs (Arvanitoyannis and Bosnea, 2004). Such interaction between food and packaging materials is considered to be an interchange among food, packaging, and the environment and can impact food quality, safety, and/or package integrity. The main goal of food packaging is to protect food from external environmental factors, but food–packaging interactions also can compromise the quality and/or safety of foods.

However, the mass transfer of additives from packaging to the foods is undesirable and can alter the food’s flavor. Other undesired phenomena include removal of some desirable flavors from the food to the packaging and the uptake or release of moisture by permeation. An interesting possibility is that food quality and safety could be enhanced via such package-to-food interactions. Recently for instance, diverse studies have been used in designing packaging with active component materials that scavenge oxygen, as opposed to acting as a simple barrier to permeation, to improve the stability of high-fat foods (Maloba et al., 1996). Packaging designed to enhance desirable interactions with the contained food are called “active packaging” (Labuza and Breene, 1989). The food and packaging interaction could be categorized into three types: migration, permeation, and sorption. Examples are the migration of contaminants or plasticizers from recycled plastic polymers, which is considered as a regulatory and safety issue, or the migration of food additives, which could enhance food quality; the permeation of different gases, such as oxygen or carbon dioxide, that may be beneficial for modified atmosphere packaging yet undesirable for carbonated beverages; and the sorption of aroma and flavor, which could change the organoleptic properties of foods. The key theories that reinforce these interactions are based on the Fickian theory of diffusion. The theoretical basis of migration, absorption, and permeation, while the interactions between polymeric packaging and aroma and flavors (Crank, 1975, Johansson, 1996).

### 1.2 Migration from packaging material to food

**Fig. 1:**
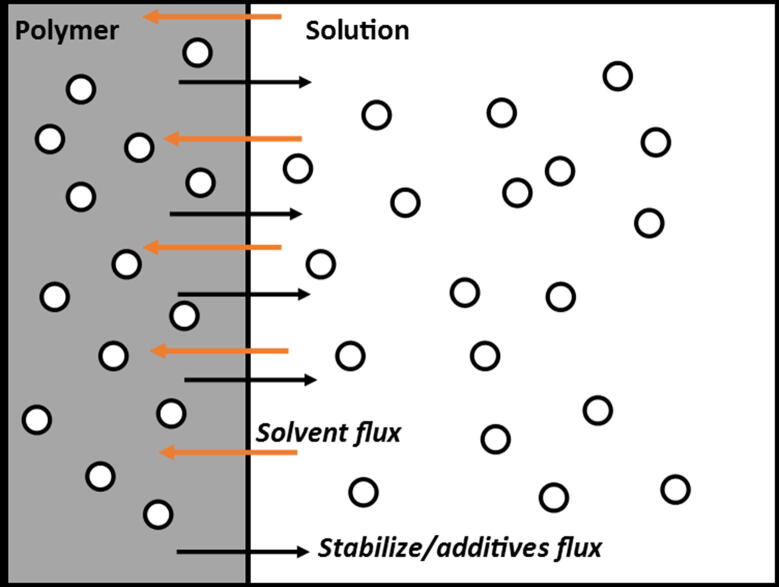
Packaging polymer and solution interface with diffusion of additives and solvent (Ferrara et al., 2001).

The migration phenomenon in packaged foods may happen in two directions simultaneously, i.e., from packaging material to the food product and vice versa (Mousavi et al., 1998). In the former case, the molecularly diffused low-molecular weight substances such as additives and oligomers from the packaging films are transferred into the foods (Helmroth et al., 2020). In the latter scenario, the mass transfer of food color, aroma, flavor, and nutrients happens from the food product to the packaging and results in a strong impact on the organoleptic properties of foods (Lee et al., 2008). The polymer packaging and food interface suggesting chemical migration is diagrammed in Fig. 1.

Migration is the transfer of chemical compounds from or to the packaging film that occurs upon contact with the food. We have considered mostly the transfer of chemical substances from packaging to food. The chemical substances can potentially come from packaging substrates (such as paper, cardboard, or plastics), but other packaging components (such as printing inks, adhesives, or coatings) could also be sources of chemical migrants. Factors that determine the extent of migration include the packaging polymer, physicochemical properties of the migrant, the food type, duration and temperature of storage, and the package-to-food proportion (because smaller packaging has a larger surface-to-volume ratio). The maintenance of food quality and safety is considered critical during the packaging process, in storage, during transportation, and in retail locations (Hron et al., 2012). Therefore, various levels of safety standards are practiced from country level (U.S. Food and Drug Administration) to regional level (European Food Safety Authority). Some certification programs, such as the Global Food Safety Initiative, have been introduced but are not yet in widespread use. Authorities have issued legislative directives about migration of chemicals into food (Arvanitoyannis and Kotsanopoulos, 2014).

Health-related risks from the materials and chemicals used in food packaging should be carefully considered and thoroughly monitored. To prevent contact and potential migration of carcinogenic chemical compounds into foods, such carcinogens need to be eliminated (Claudio, 2012). Trace metals, one of the potential sources that can contaminate food products, may enter food chains from soil; agrochemicals; water used in food processing; food-processing equipment, containers, and utensils; and from packaging.

Hazards related to the presence of trace metals in food has raised widespread health concerns. Chronic and acute symptoms including dizziness, nausea, diarrhea, vomiting, loss of appetite, sleeping disorders, and reduced conception rate may be indicative of heavy-metal toxicity. Trace metals have also been linked to cardiovascular ailments, suppressed growth, neurological and immune-system disorders, impaired fertility, increased spontaneous abortions, and higher death rates among infants (Yüzbaşi et al., 2003).

### 1.3 Mechanism of migration

Substances migrating from food packaging to foods are highly complex. Diffusion phenomena are the main mechanism of migration where the macroscopic mass movement of molecules occur from higher to lower concentration gradients until an equilibrium is reached (Miltz et al., 1997, Simoneau, 2008). The rate of molecular diffusion is shown mathematically by Fick’s second law:

$$\mathrm{dCp}/dt=D(d^{2}Cp/dx^{2}),$$

where

*Cp:* concentration (mg/g) of migrant in packaging material
*D:* coefficient of diffusion (cm^2^/s)
*t(s):* time of diffusion
*x:* distance (cm) between food and packaging material (Silva et al., 2007).

Although the mathematical models are under continuous development, their reliability is appreciable for measuring contamination from packaging chemicals. A complete understanding of the factors influencing the migration is well suited to improving quality control by determining the variables with the greatest impact. Such improved evaluation of chemical migration from package to food would help limit and control food contamination and improve food safety.

### 1.4 Types of migration

#### 1.4.1 Migration according to number of migrants

There are two terms used for migration that should be not confused, overall migration and specific migration. Overall migration refers to the sum of the mass transfer of all releasing substances from a unit area of packaging material, and specific migration refers to the migration of a particular chemical species (Robertson, 2006). Both types of migration are considered important based on analytical objectives.

#### 1.4.2 Migration related to foods nature

Migration can be divided into three categories related to food systems–nonmigrating, volatile, and leaching system. In a nonmigrating system, very little mass transfer of pigments or some inorganic substances occurs as compared to the high molecular weight of packaging polymers. On the other hand, in a volatile migrating system, minor volatile aromatic compounds transfer to the package even without direct contact between the food and the packaging material, though contact could improve such migration. This type of migration is considered in dried products where less direct contact occurs between food and packaging material. Under such conditions the volatile substances migrate in three stages: diffusion or evaporation of migrant, desorption from a product, and adsorption onto the product. However, for a leaching type of migration system, the food must contact the packaging for the migrant transfer to occur. In this system, the mass transfer of a migrant is initiated with its diffusion from the package material, is followed by dissolution, and ends with dispersion into the food product. A common example of this system is the mass transfer of substance to fluid or semisolid foods from daily-use plastic packaging materials upon direct mutual contact (Lee et al., 2008).

#### 1.4.3 Migration based on coefficient of diffusion

The process of diffusion is the key determinant of the rate of diffusion, but diffusion estimation becomes challenging when the package is in contact with the food, which may alter the diffusion rate in the packaging material. This migration could be categorized into three clearly distinguishable categories. In the first category, the diffusion coefficient approaches zero, and thus there is a minimal migration potential. In the second category, the diffusion coefficient possesses a constant value and experiences no impact from the food component or storage time. However, in the last category, the diffusion of a substance remains insignificant unless the food is in direct contact with the packaging material (Aurela, 2001).

#### 1.4.4 Contact migration

In this category, as the name suggests, the migration of a substance happened from the packaging to the food only upon contact. For example, the transfer of additives from the cardboard pizza box to the pizza or transfer of monomers and plasticizers from a plastic tray, pouch, or wrapping to the foods (Karen et al., 2006).

#### 1.4.5 Gas-phase migration

In this type of migration, the substance permeates from the outer coating or printed layer of the package to the inner layer of the packaging material. The mass transfer of a particular substance happens through the medium of gas (Karen et al., 2006).

#### 1.4.6 Penetration migration

In penetration migration, a substance from the outer coated or printed layer of the packaging material migrates toward the inner layer or contacting side of the packaging material through the packaging material itself. The substance upon reaching to inner side of the package could migrate to the contained food either by contact or by gas-phase migration (Karen et al., 2006).

#### 1.4.7 Set-off migration

This type of migration is related to the mass transfer of inks, varnishes, and coatings from the outer printed side to the inner side of the packaging films by stacking (e.g., of printed cartons) or during reeling (e.g., winding printed wrappers into a reel). The set-off migration could be either visible or invisible depending on the specific substance. Substances clinging to the inner side by set-off migration could easily transfer either by gas-phase migration or by direct contact and could contaminate the packaged or wrapped food (Karen et al., 2006).

#### 1.4.8 Condensation/distillation migration

Although heat treatment of foods is used to improve their shelf stability, the transfer of substances may happen during processes of boiling or sterilization of pouched food or food in trays or cartons. Typically, the volatile components from the packaging or from distillation of moisture from steam released from aqueous foods migrates from package to food and vice versa (Karen et al., 2006).

### 1.5 Factors influencing migration phenomenon

Given the complexity of migration phenomena, several factors could affect the process. The extent and the rate of migration is variously influenced. The primary factors include the following:

#### 1.5.1 Nature of foods

The nature and composition of the food are critical factors in migration evaluation. For example, foods with surplus fats reportedly display high levels of migration (Triantafyllou et al., 2007). Various food simulants have already been used to study the influence of food nature on migration. Many studies have been conducted to investigate the mass transfer of substances between packaging and food by applying solubility parameters that helped test the extent of migration during food production in real time. In this regard, different food simulants are recommended by different authorities in Europe and the U.S. (Table 2).

**Table 2:** Listing of common food simulants used for migration testing (Franz, 2000, Rossi, 2000).

Solvents used for migration testing	Simulant category
Distilled H_2_O	Simulant A
Aqueous acetic acid (3% w/v)	Simulant B
Aqueous ethanol (15% v/v)	Simulant C
Sunflower oil or rectified olive oil	Simulant D

#### 1.5.2 Type of contact

Numerous studies have indicated that migration levels are associated with the type of contact (direct or indirect) between the food and the packaging. Specifically, direct contact between food and the packaging enhances the mass-transfer rate, and with indirect contact, the gas medium between the food and the packaging results in relatively slower migration (Anderson and Castle, 2003).

#### 1.5.3 Duration of contact

Mass transfer of specific substances of concern is largely dependent on the duration of contact of food with the package. Experimental data has shown that the mass transfer of a substance is proportional to the square root of the duration of contact between the food and packaging material (Arvanitoyannis and Bosnea, 2004). Other experimental evidence has shown that the log of the duration of equilibrium of a migrating substance is inversely correlated with temperature (Poças et al., 2011).

#### 1.5.4 Temperature of contact

The rate and extent of migration are directly influenced by the temperature of food at storage. At higher temperatures, migration rates increase as the equilibrium is rapidly established between the packaging headspace and the food (Triantafyllou et al., 2005).

#### 1.5.5 Nature of packaging material

The packaging material has a significant impact on the migration of a substance. Typically, the thickness and the plasticization of the packaging material affect the migration of specific additives. Thicker packaging slows migration, whereas thinner packaging allows greater migration (Nerin et al., 2007). However, the presence of recycled additives and ingredients did not present any discernible correlation with migration rates (Poças et al., 2011).

#### 1.5.6 Migrant characteristics

The nature of a migrating substance (or potential migrant) have significant impact on the migration extent and rate. Mass transfer of a highly volatile substance happens at a greater pace. However, substances with relatively higher molecular weights exhibit lower migration rates (Johns et al., 2000). The microstructure of the migrating substance also impacts its migration level. More specifically, the configuration of the migrating molecules (e.g., spherical vs branched and with or without side chains) affects migration differently; for instance, branched molecules exhibit lower migration rates (Maloba et al., 1996, Triantafyllou et al., 2005).

#### 1.5.7 Migrant concentration in packaging

Obviously, mass transfer of a migrating species occurs at a higher rate from the packaging to the food based on its concentration in the packaging material. It is also evident that a higher amount of migrants is found in the food matrix after a given time of storage under experimental conditions (Mariani et al., 1999).

### 1.6 Types of food packaging migrating compounds

#### 1.6.1 From printing inks

The packaging, besides providing containment for the foods, also delivers information about the brand and composition and provides nutritional labelling for the foods. High-performance plastic packaging materials are very effective for shelf stability of the product until expiry. Generally, the single layer of material used in packaging the food products also has printed inks to disseminate the product description to consumers. A food stored in such packaging could increase the probability of transfer of printing dyes or inks to the food and thus may pose a quality and safety challenge. Printable ultraviolet (UV)-curable inks and varnishes are commonly used in packaging and normally comprise three components: a monomer, an initiator, and a pigment. For application, the ink is exposed to a UV source where the photoinitiator is converted into a free radical that ultimately reacts with the added monomers and starts polymerization (Castle et al., 1997, Robertson, 2006, Samonsek and Puype, 2013). During polymerization, the developed polymers bind the base polymeric packaging irreversibly and entrap the pigments resulting in a fast and good-quality printed surface. Some other printing inks are composed of pigmented resins and an organic carrier or polar solvent. This type of ink requires adequate drying if solvent removal is necessary, and print quality is highly dependent on numerous factors. In the case of UV-cured inks, the unbalanced formulation of the monomers and photoinitiators and incorrect functioning of the UV source may result in excessive residuals of monomers or photoinitiators. Thus, a potential migration of these substances into a food matrix would alter the organoleptic properties of food and compromise the safety of the food. Additionally, the interaction of the migrating species with the food would initiate taints and possibly result in loss of quality and nutritional value (Johns et al., 2000, Boon, 2008, Bradley et al., 2013).

Migration of benzophenone, a frequently used odorless photoinitiator, has been reported to generate alkyl benzoates, which contribute to undesirable flavors. Studies have reported the presence of printing inks in snacks and confectionary products well above the minimal detectable limits. Similarly, plasticizers, commonly used in packaging materials and in printing inks to provide functions such as flexibility, wrinkle resistance, and adhesion, are capable of contaminating foods by migrating from the packaging films. The presence of phthalates and other compounds such as tris(2-ethylhexyl) trimellitate, sulphonamides, and N-ethyl-toluene and N-methyl-toluene has been detected in printing inks. However, the chance of mass transfer of printing ink is relatively lower than that of the plasticizers used in the fabrication of packaging materials during direct contact with foods (Rasff, 2005, Boon, 2008, Bradley et al., 2013).

#### 1.6.2 From adhesives

Adhesives are the compounds that are used to seal the packaging and they can also migrate to the foods during packaging or storage. The adhesives commonly used in the packaging industry are hot-melt, cold-seal, pressure-sensitive polyurethanes and acrylics that are water- or solvent-based or solvent-free. The selection of adhesives must be based on the type of packaging and characteristics of the food product. For example, the use of a hot-melt adhesive is inappropriate for wrapping bars of milk chocolate. Also, special requirements apply in cases where aromatic volatiles are directly incorporated in cold seals to augment the food-product perception at the time of opening (Athenstädt et al., 2012, Sella et al., 2013).

From a previous survey by adhesive manufacturers, a listing of more than 360 substances was compiled to indicate potential chemical migrants from adhesives into foods (Hoppe et al., 2016). A subsequent study focused on the chemical composition and level of migration of polyurethane-based adhesives. The migrating residuals (e.g., polyether, polyols, and cyclic reaction products derived from polyester polyols) were identified at concentrations of 10–100 μgdm^−2^ (Sella et al., 2013, Hoppe et al., 2016).

The migrants from the inks of a printed packaging surface also can easily transfer to the layer of adhesives, especially when the packaging is stacked, and thus could ultimately migrate to the food matrix during the process of packaging. However, in the case of multilayer packaging systems such as laminates, the chances of potential contact migration of migrants are increased significantly. The multilayer laminates are complex packaging materials that are manufactured by layering of different polymeric with non-polymeric materials (e.g., metals) to achieve particular packaging characteristics. The existence of diverse components along with adhesives could greatly increase the likelihood of health problems while also making the identification and detection processes more difficult and complex (Athenstädt et al., 2012, Sella et al., 2013, Hoppe et al., 2016).

### 1.7 Plastic packaging

#### 1.7.1 Plasticizers

Most plasticizers are the esters of phthalic (phthalates) and adipic acids. Dioctyl phthalate, di-2-ethylhexyl phthalate and di-2-ethylhexyl adipate are systematically applied during the preparation of packaging material (Rahman and Brazel, 2004). The phthalates are cast off in sealing gaskets and cap-sealing resins for bottled food, polyvinylchloride (PVC) films, and some plastic packaging. Phthalates once used as plasticizers in polymeric packaging films are characterized by low molecular weight, thus facilitating the package-to-food migration. Numerous studies have reported plasticizers as potential migrants that could transfer to foods from the packaging (Pedersen et al., 2008).

#### 1.7.2 Thermal stabilizers

Thermal stabilizers are commonly incorporated in plastic materials, including PVC and polystyrene (PS) (Lau and Wong, 2000). Generally, epoxidized seed and vegetable oils (e.g., soybean oil–esterified soybean oil) is commonly used in a wide range of food-contact plastic-polymer films as heat stabilizers, lubricants, and plasticizers (Lau and Wong, 2000) From studies of the impact of the degree of purity on toxicity, it was found that residual ethylene oxide is highly toxic (Food Standards, 2012).

#### 1.7.3 Slip additives

Fatty acid-based amides are extensively used as additives in plastic packaging manufactured from polyolefins, PS, and PVC. Slip additives, which are directly incorporated into the plastic formulations, cause the emergence of surface bloom. These compounds are used to impart specific characteristics to the products. For example, they provide lubricating properties to the packaging materials to avoid sticking or conglomeration and also to reduce static charges (Cooper and Tice, 1995, Arvanitoyannis and Bosnea, 2004).

#### 1.7.4 Light stabilizers

These chemicals are used in plastic packaging materials (polyolefins) to enhance endurance for long-term applications. Light stabilizers are used in many applications to improve long-term weathering properties of plastic polymers such as polyolefins. Polymeric hindered amines (e.g., Chimasorb 944 and Tunuvin 622) are widely used in polyolefins as light stabilizers (Poças and Hogg, 2007, Grob, 2002). These amines are detected through sophisticated analysis based on ultra-performance liquid chromatography with detectors of dual wavelengths (UV and visible). The procedure provides dependable results, offering a chance to develop functional tools that could help verify compliance with legal limits (Noguerol-Cal et al., 2010).

#### 1.7.5 Antioxidants

When polymers are exposed to UV light and air, they could be degraded significantly owing to the oxidation reactions. Antioxidants can be applied to decrease the degree of oxidation and enhance stabilization of the polymers. Tinuvin P, Tinuvin 776 DF, Tinuvin 326, Tinuvin 234, Irganox168, Irganox 1010, Irganox 1330, and Irganox P-EPQ are the commonly used chemical antioxidants in plastic packaging materials (Nestmann et al., 2005). Also, vitamins such as A, C, and E and derivatives such as tocopherols, tocotrienols, and carotenoids can be added. Similarly, some metal ions (e.g., selenium) are crucial for the activity of antioxidant enzymes, and other phytochemicals, such as CoQ10, glutathione, and lipoic acid, are also considered good in controlling the oxidation of packaging materials. Additionally, mass transfer of synthetic antioxidants, such as butylated hydroxyanisole, butylated hydroxytoluene, tertiary butylhydroquinone, and propyl gallate have been reported to transfer between food matrix and packaging materials (Papas, 2012)

#### 1.7.6 Solvents

Various solvents are used in the preparation of solutions or in dispersions of the printing inks used in plastic packaging. The solvents are mainly low-molecular-weight organic compounds such as ethers, esters, alcohols, and ketones. These solvents are mostly evaporated from printed plastic packaging but may also disperse by distillation, penetration, or direct contact (Boon, 2008). However, some residue of the base solvent may remain entrapped in the packaging materials and later get transferred to the food upon direct contact or after release into the packaging headspace. The amount of solvent transferring to the food from packaging material is highly dependent on the concentration and distribution of the solvent (Robertson, 2006). Therefore, potential migration of residual solvent may pose a risk of changing the food organoleptic properties.

#### 1.7.7 Monomers and oligomers

Many monomers and oligomeric building blocks connect to produce polymers by various chemical reactions. Styrene is among monomers that are widely applied to produce PS, which is used in packaging that is in direct contact with foods. PS is used mostly as containment for a range of dairy products (ice cream, cottage cheese, yogurt), fruit juice and other drinks, poultry and other meat, bakery products, and fresh produce (Tawfik and Huyghebaert, 1998). Leibman (1975) reported that a styrene monomer may degrade into its respective oxide, which is characterized as a severe mutagenic and if metabolized in body can produce hippuric acid that could be excreted from the body in urine. Styrene exposure could result in organ toxicity and irritation of the skin, eyes, and lungs with simultaneous suppression of the activity of the central nervous system. Also, Tang et al. (2000) reported that the average identified level of styrene monomers in food packaging is 100–3000 ppm.

#### 1.7.8 Isocyanates

Isocyanates are commonly used to produce polyurethanes and are used in some adhesives for the preparation of food packaging. Also, aromatic amines, especially primary amines, are a subcategory of this class of compounds, and Miltz et al. (1997) reported their migration into foods from materials such as rubber, epoxy polymers, aromatic polyurethanes, and azo dyes. The toxic effects of isocyanates on human health have been extensively reviewed in other studies (Lau and Wong, 2000). The maximum level of isocyanates residues must be < 1.0 mg kg^−1^ in the final packaging material. However, only 12 isocyanates are approved for use in food packaging.

#### 1.7.9 Vinyl chloride

Under normal temperature and pressure conditions, vinyl chloride is a colorless gas. It is compressed into liquid under high pressure and has been used in the preparation of polyvinyl chloride-based packaging materials (Robertson, 2006). Vinyl chloride can leach from PVC bottles and food packaging and may modify the food organoleptic properties and also may result in toxicity. Because it is highly toxic, maximum allowed levels in food packaging have been in place since the 1970s (Castle et al., 1996). The Agency for Toxic Substances and Disease Registry (2006), a U.S. government agency, reported that records show the daily dietary exposure to vinyl chloride was <0.0004 μg kg^−1^ in the United States and United Kingdom in the 1970s and early 1980s. Many organizations, including the U.S. Food and Drug Administration, have established limitations regarding the maximum vinyl chloride content in food-packaging films and bottles.

#### 1.7.10 Acrylonitrile

The monomer acrylonitrile (AC) is used extensively as starting material in the production of plastics, resins, elastomers, and synthetic rubbers (National Industrial Chemical Notification and Assessment Scheme, 2000). It is also found in diverse polymeric materials used in manufacturing food packaging. For example, terpolymer consists of three or more AC monomers in combination with styrene and butadiene. AC/butadiene/styrene resins can be used as food-contact materials. The relative amounts of the resins used in the polymers may vary depending on different specific characteristics necessary for different products. However, AC monomer is toxic; Lickly et al. (1991) examined and evaluated the association of its residues in polymers by using various food simulants.

#### 1.7.11 Polyethylene terephthalate oligomer

Polyethylene terephthalate (PET) oligomers are used mainly in manufacturing of trays and bottles for packaging of various types of food (including fresh produce) and drink (including mineral water, juice, beer, carbonated beverages, and milk). It is a thermoplastic polyester produced by a condensation reaction (esterification) of ethylene glycol in the presence of terephthalic acid or its derivative as dimethyl terephthalate (Kim and Lee, 2012). PET is easy to mold for producing trays and dishes of various desired shapes, and due to its temperature resistance (∼220 °C), these containers can be used in heating or reheating of food. However, PET reportedly contains small amounts of low-molecular-weight oligomers (some dimers to pentamers). Additionally, the main volatile substance found in PET is acetaldehyde, which is of high significance owing to its effects on food odors, especially in cola-type beverages. Lau and Wong, 2000) detected these cyclic chemical substances in various beverages at levels of 0.06% and 1.0% depending on the type of PET (Nerín et al., 2013, Silano et al., 2008).

### 1.8 Metal packaging

#### 1.8.1 Tin

Tin-based cans are used in containing foods and various carbonated and noncarbonated drinks. Tin traces transfer into the foods contained in tin cans with or without any lacquering. Foods with higher concentrations of tin (e.g., ∼500 mg kg^−1^) reportedly can cause severe gastrointestinal ailments (Omori et al., 1973, Benoy et al., 1997). According to clinical trials, Boogaard et al. (2003) found that the threshold for an acute effect from tin starts after consuming a dose >730 mg kg^−1^. A thin layer of tin can help protect corrosion of metal cans. Although usually no lacquering is done for tin, especially when oxygen scavenging is desired, a lacquer coating is otherwise preferable because an uncoated can may lead to various interactions between the tin and the food matrix (Oldring, 2007).

#### 1.8.2 Lead

Despite its toxicity and although it is known to be a common contaminant in foods, lead is commonly used in metal food and beverage containers. Lead toxicity could damage the central nervous system and has negative impacts on various body organs in humans. Infants are especially prone to lead toxicity because of the greater retention of lead in their brains and bones. Even a subacute consumption of lead could result in mental retardation, convulsions, and encephalopathy in children (Skrzydlewska et al., 2003, Robertson, 2006).

#### 1.8.3 Aluminum

Al is used in preparation of laminate or multilayer food packaging or directly design cups and trays. It is used mostly in alloy form with other metals (such as Cu, Zn, Si, Mn, Mg, and Fe) to design food packaging. Small concentrations of Al are found in various plants and animals (Taylor, 1964). Unlike so many other vital elements that take part in the metabolism of animals, Al is not essential for the functionality of enzymes or any other metabolic process. High intake and increasing levels of Al in tissues have been associated with many disorders (such as dialysis encephalopathy, osteodystrophy, and microcytic anemia). Other than the recommended-maximum-dose Al intake from food and beverages, Al also migrates from cooking utensils and from storage or packaging. Because pure Al cannot be used to produce packaging materials, alloys of Al with Fe, Ag, Cu, Mn, and Zn are used instead. Therefore, elements other than Al could be present in foods upon corrosion of the cans used to contain the food (Rodushkin and Magnusson, 2005, Robertson, 2006).

#### 1.8.4 Chromium

Electrolytic Cr coating is widely used as a thin layer in tin-based cans to make them more stable against oxidative damage and to strengthen enamel adherence. Cr is characterized by relatively high toxicity and undesirable sensory properties. Also, in its hexavalent form (Cr(VI)), it could have a severe impact on living organisms owing to its having both carcinogenic and mutagenic properties (Skrzydlewska et al., 2003, Kim et al., 2008).

### 1.9 Paper packaging

#### 1.9.1 Dioxins

These form a class comprising a large number of synthetic polychlorinated compounds that include but are not limited to polychlorinated dibenzofurans and dibenzo-dioxins. Dioxins are used in paper-based packaging for food applications. Dioxins are reported as highly toxic and mutagenic organic compounds. The isomer called 2,3,7,8-tetrachlorodibenzo-*para*-dioxin is the most toxic among all the dioxins (Ackermann et al., 2006).

#### 1.9.2 Benzophenone

This organic compound is used in inks and lacquers as a photoinitiator and also is used as a wetting agent for dyes and pigments to improve their flowability. In general, 5%–10% of this compound is used once considered as photoinitiator in inks (Anderson and Castle, 2003). UV light is used to cure the printing inks for cardboard packaging thus online production process of finished packaging is relatively faster. However, because the benzophenones used in these inks may not get totally removed during this process, benzophenone could migrate to the inner sides of the cardboard components during stacking before forming the cardboard cartons or boxes. Also, the use of fiber recycled from cardboard may increase the probability of the presence and migration of benzophenones. The specific compound 4-methoxybenzophenone is also used but reportedly is carcinogenic and mutagenic (Muncke, 2009).

#### 1.9.3 Nitrosamines

Nitrosamines are commonly found in foods and beverages (Robertson, 2006). These amines are considered potential carcinogens and genotoxic. Nitrosamines are formed endogenously in the human body by reaction of amines with salivary nitrates or nitrites (Tricker and Preussmann, 1991). Nitrosamines could also come from waxed cardboard and paper. These materials contain morpholine and N-nitrosomorpholine, which contaminate food after migration from a surface upon contact during storage and the processes involved in packaging.

#### 1.9.4 Chlorophenols and chloroanisoles

Chlorophenols are organochlorides that have been industrially used for the production of biocides, fungicides, and herbicide intermediates (Kirwan et al., 2011). These compounds commonly transfer into food from packaging materials. Contamination of foods with these organochemicals results in the production of off-flavors and taints (Jelén, 2006).

### 1.10 Glass containment

Chemical glass is resistant to water or water-based solutions and organic substances. Acidic solvents have very limited impact on the silica component, although other ingredients of glass could be attacked by these solvents. Autoclaving of glass within various solvents resulting in the leaching of traces of alkali and silica has been thoroughly investigated. However, this has almost no impact on the organoleptic properties of the foods. Similarly, minimum contamination of foods is reported for cadmium and lead, as these metallic components are rarely present in glass containers designed for food packaging. Although the rate of glass recycling has greatly increased, the amount of chemical migration in glass containment is still very low (Shaw, 2013).

### 1.11 Additive derivatives and monomers

Other than the multiple above-mentioned types of possible food contamination, various derivatives of additives and monomers also could transfer to foods. In particular, direct contact between food and packaging material could result in migration of chemical substances and potentially contaminate the product. The environment also could contaminate the food if water and air quality are not properly monitored and thoroughly cleaned (Lau and Wong 2000).

### 1.12 Benzene and other volatiles

For diverse food-contact plastics, organic components such as benzene or alkyl-benzene are typically produced at higher temperatures. For example, benzene is known to migrate into food from PET-, PVC-, and PS-based food packaging. Owing to its low molecular weight, it can easily diffuse through the package and contaminate foods. Therefore, the detection of benzene levels in plastic-based food packaging is necessary given its potential carcinogenicity (Anderson and Castle, 2003, Arvanitoyannis and Bosnea, 2004).

### 1.13 Environmental contaminants

The surrounding environment could be a major source of food contamination if it is not hygienic. Numerous environmental contaminants, such as dust, microbes, insects, and naphthalene, can be transferred into foods and result in contamination. This may occur through damaged or absorbent packaging material with subsequent migration to the foods (Raloff, 2000). For example, concentration of naphthalene could rise significantly in the environment where naphthalene-based insect repellants are in use. Similarly, milk or milk-based drinks packaged in low-density polyethylene containers have shown increased concentration of naphthalene once stored in high-naphthalene environments. Also, during processing and supply cycles, the risks of packaging and hence food contamination may increase. Similarly, hydrogen peroxide, a widely applied sanitizer used in sterilizing polypropylene and polyethylene aseptic food packaging, could be a contaminant (Lau and Wong, 2000).

### 1.14 Other contaminants

Besides the already-mentioned contaminants, there are various possible components that could migrate and contaminate foods. For instance, PVC-based food packaging contains the contaminant dioxin. Similarly, benzene, diphenyl thiourea (a heat-stabilizing agent) (Griffith, 1989), processing-aids additives (Satyanarayana and Das 1990), and diverse volatiles may migrate into packaged foods. Contamination of foods by diphenyl thiourea and its derivatives (e.g., aniline, diphenylurea, isothiocyanatobenzene) reportedly has been found in packaging materials (Lawson et al., 1996, Careri et al., 2002, Arvanitoyannis and Bosnea, 2004).

### 1.15 Conclusion and future outlook

For a specific food product, a careful choice of packaging material should be made by considering the end-product components and all their possible interactions as well as the resultant impact on food quality and safety. For any food-packaging selection, the benchmark is compliance with valid legislation and regulations, which may demand measurement of global and specific migration to assess the safety of the packaging material. The potential for taints migration should be estimated by considering the following:

1. Is the packaging material optimized to reduce the chances of potential migration of available components?
2. The probability of migration of any potentially migrating component into the packaged food depends on the food composition, which determines the affinity of migrants toward the model food. For instance, the majority of migrating constituents that result in taints production includes hydrophobic elements, which pose serious challenges in packaging for high-fat foods.
3. The impact of the migrating compounds on the organoleptic properties of foods is affected by the flavor intensity of the foods. Thus, the extent of tolerated migration (within legislative limits) also should be according to the flavor characteristic of the foods.

## Declaration of Competing Interest

The authors declare that they have no known competing financial interests or personal relationships that could have appeared to influence the work reported in this paper.
